# Mechanosensitive Piezo1 channel: an emerging target in demyelination disease

**DOI:** 10.3389/fncel.2025.1556892

**Published:** 2025-07-09

**Authors:** Yuxi Zhang, Xiaoke Yang, Simin Deng, Chenxu Wang, Jialing Hu, Qinghai Lan

**Affiliations:** ^1^Department of Anesthesiology, Ganzhou People’s Hospital, Ganzhou, China; ^2^Jiangxi Medical School, Nanchang University, Nanchang, China; ^3^Department of Emergency Medicine, The Second Affiliated Hospital of Nanchang University, Jiangxi Medical College, Nanchang University, Nanchang, China

**Keywords:** Piezo1, demyelination, demyelinating diseases, multiple sclerosis, myelsin

## Abstract

Many physiological processes in the human body are initiated by mechanical signals, which are transmitted via ion channels. Piezo-type mechanosensitive ion channel component 1 (Piezo1) is a protein highly expressed in the brain, playing a critical role in sensing changes in the mechanical microenvironment. Extensive research has demonstrated that Piezo1 is an essential component for generating currents in mechanically activated cation channels. It is involved in several key processes in the nervous system, including neuronal development and differentiation, nerve regeneration, axon guidance, and myelination. Demyelinating diseases, characterized by the loss of nerve myelin sheaths, occur in the central or peripheral nervous system. These diseases are clinically challenging due to their diverse etiologies, multiple types, poor prognosis, and lack of definitive cures. This article aims to review the current research on the role of Piezo1 in myelination and its involvement in demyelinating diseases, as well as to explore the potential of targeting Piezo1 for therapeutic interventions in such conditions.

## 1 Introduction

### 1.1 Mechanical force

Mechanical forces are crucial to human health and play a significant role in the cellular pathology of diseases. These forces drive numerous processes, including touch and hearing perception, blood pressure regulation, and organ development. Sensory experiences such as touch and environmental sounds are transduced into mechanical signals within the extracellular matrix (ECM) of human cells (Coste et al., 2010; Beech and Kalli, 2019). Mechanical signals influence the activity of the nervous system through mechanical interactions between neural cells and surrounding tissues. Forces generated by neighboring cells, fluid flow, or large-scale tissue movements interact with neural cells, transmitting mechanical signals to neurons (applying mechanical pressure to neurons), which supports neuronal growth (Wan et al., 2021; Engler et al., 2006; Keung et al., 2012; Pillai and Franze, 2024; Xu X. et al., 2021).

### 1.2 Mechanical transduction and mechanosensitive ion channels

In addition to providing mechanical support to direct mechanical interactions between cells and tissues, ECM drives biological signal transduction, and its mechanical signals can be converted into intracellular signals, thus participating in the regulation of cellular physiological activities (Saraswathibhatla et al., 2023). Mechanical transduction (cell-ECM mechanical transduction), the process of converting mechanical stimuli into biological signals, is mediated by mechanosensitive ion channels (MSCs). In 1979, Corey and Hudspeth first recorded electrical currents activated by mechanical signals, and the study of MSCs has increased since then. These channels translate mechanical signals from the ECM into intracellular chemical or electrical signals, these signals can cause cytoskeletal rearrangement or activate intracellular signaling cascades that ultimately lead to changes in gene expression (Lam and Calvo, 2018). When MSCs are mechanically stimulated to open, corresponding ions enter and exit the cell and generate currents. For example, increased calcium influx from mechanosensitive ion channel TRPV4 activation drives nuclear localization of RUNX2, a transcription factor involved in osteogenic differentiation. As to how MSCs change from closed to open when mechanically stimulated, membrane tension model and tether model have been proposed to explain this problem. The membrane tension model is based on the principle that mechanical forces applied to the membrane lipid bilayer generate membrane tension on the portal channel, while the tethered model is based on the principle that forces applied to the cell or extracellular matrix are transmitted to the portal channel through tethered junction channels and outer membrane components (Jin et al., 2020; Kefauver et al., 2020).

In recent years, many progresses have been made in the field of MSCs. The mechanoreceptor channel families found include epithelial sodium channel/denaturing protein (ENaC/DEG), transient receptor potential channel (TRP) and two-pore domain potassium channel (K2P). However, Coste et al. discovered the PIEZO ion channel gene family in 2010, which is the most important breakthrough in the field of mechanical transduction. The Nobel Prize in Physiology or Medicine in 2021 was awarded to Professor Ardem Patapoutian (Joint). The discovery of PIEZO channel greatly promoted the development of mechanical transduction field (Otero-Sobrino et al., 2023; Delmas et al., 2022). PIEZO family is a family of mechanically activated cation channels discovered by Coste et al. in eukaryotes, including PIEZO1 and PIEZO2. PIEZO channels are a class of non-selective Ca2^+^ cation channels, which are evolutionarily conserved membrane proteins with triple helix structure. PIEZO1 is widely expressed in non-sensory tissues of higher vertebrates, such as the lungs, bladder, skin, and gastrointestinal tract, where its expression levels are notably high (Xu X. et al., 2021; Zhou and Martinac, 2023; Li et al., 2022; Swain and Liddle, 2023; Wu et al., 2017; Cahalan et al., 2015). PIEZO1 is involved in various critical physiological processes, including cardiovascular regulation, embryonic development, and urinary osmoregulation (Kefauver et al., 2020; Lei et al., 2024; Fang et al., 2021; Bryniarska-Kubiak et al., 2021).

**Figure 1:**
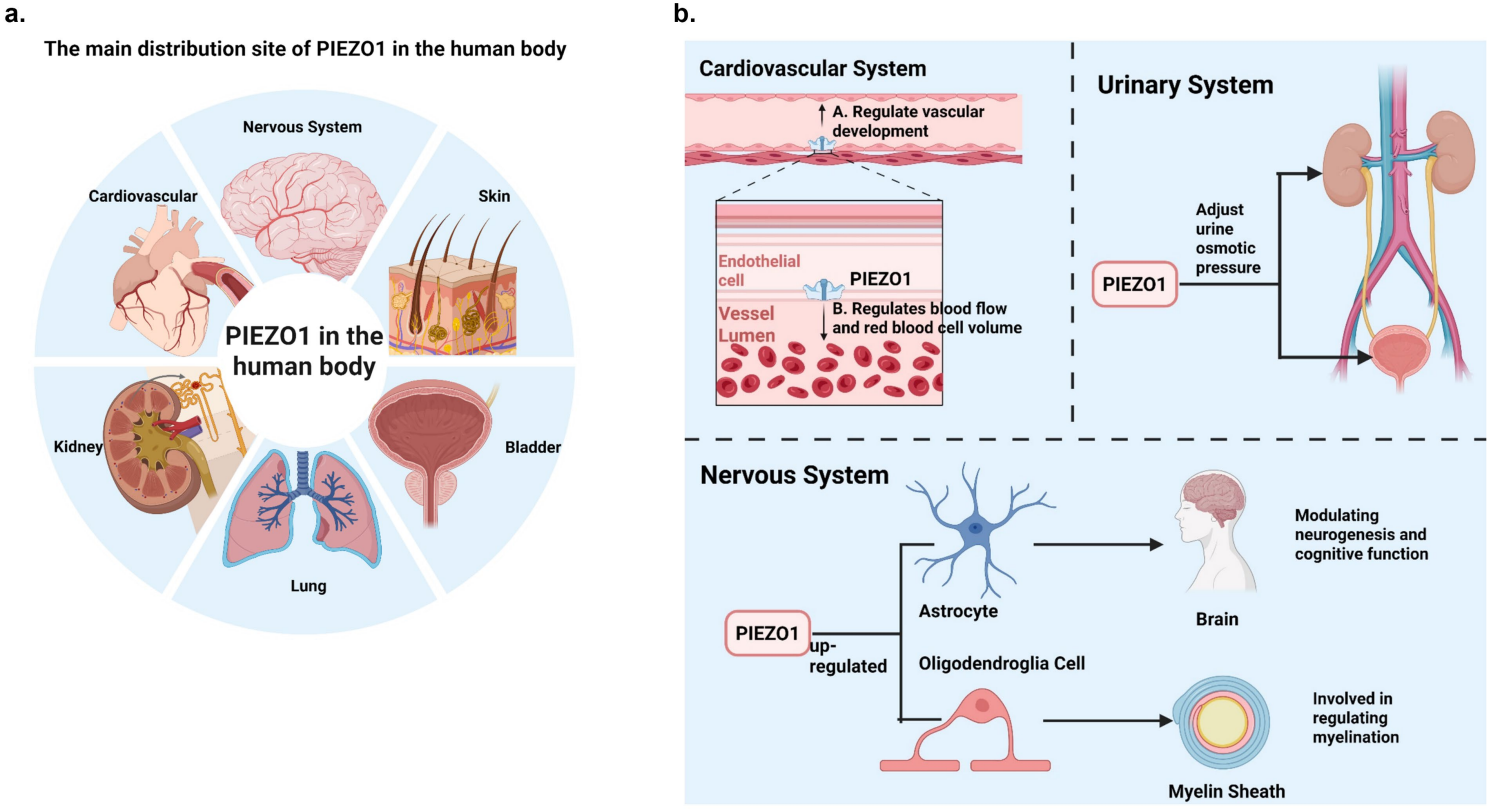
**(a)** The main distribution site of PIEZO1 in the human body. PIEZO1 is widely distributed in non-sensory tissues of higher vertebrates, such as lung, kidney, bladder, skin and cardiovascular tissues, and PIEZO1 expression levels are relatively high. In addition, PIEZO1 is distributed in the nervous system. **(b)** PIEZO1’s main physiological role in humans. This figure briefly summarizes several physiological effects of PIEZO1 in the human system (cardiovascular system, urinary system and nervous system).

PIEZO1 also plays a vital role in the nervous system. Increased PIEZO1 expression has been linked to enhanced learning and memory. Chi et al. (2022) demonstrated that astrocytic PIEZO1-mediated mechanical transduction regulates adult neurogenesis and cognitive functions, loss of PIEZO1 in astrocytes resulted in significant reductions in hippocampal volume and brain weight, and long-term potentiation (LTP) function was impaired in PIEZO1 knockout mice. In contrast, overexpression of PIEZO1 in astrocytes substantially enhanced mechanical conduction, LTP, and learning and memory performance. PIEZO1 also plays an important role in oligodendrocyte-mediated myelination. During myelination, PIEZO1 on the axon surface of neurons senses the mechanical forces generated by oligodendrocytes surrounding axons when oligodendrocytes begin to surround axons and participates in regulating myelination. Therefore, PIEZO1 also plays a role in nervous system-related myelination pathologies (Zheng et al., 2023). In Alzheimer’s disease (AD), PIEZO1 activation in microglia and astrocytes helps clear characteristic pathological markers such as amyloid-beta plaques (Jäntti et al., 2022; Velasco-Estevez et al., 2018). Research by Li et al. (2022) has shown that PIEZO1 channels determine the mechanosensitive lineage of neural stem cells, as inhibiting PIEZO1 expression in these cells suppresses neuronal growth. Furthermore, Harraz et al. demonstrated that PIEZO1 expression in central nervous system (CNS) capillaries plays a role in regulating cerebral blood flow (Martín-Durán and Hejnol, 2021). PIEZO1 is also implicated in sensory neuronal pain (Xu X. et al., 2021) (see Figure 1).

Neural fibers consist of axons surrounded by myelin sheaths, which are lipid membranes produced by oligodendrocytes in the CNS and Schwann cells in the peripheral nervous system (PNS). Based on appearance and function, the vertebrate nervous system is divided into gray matter (composed of cell bodies and dendrites) and white matter (composed of axons) (Simons and Nave, 2016; Bakhti et al., 2011; Salzer, 2015).

### 1.3 Brief description of demyelinating diseases

Demyelinating diseases, which result from damage to myelin sheath or dysfunction of myelin-forming cells, are a significant global health burden. These diseases have diverse etiologies and are categorized into CNS and PNS demyelinating diseases. CNS demyelinating diseases include inflammatory, viral, metabolic, ischemic-hypoxic, and compressive types (Love, 2006). Such diseases impair the brain, spinal cord, and optic nerves, leading to various symptoms affecting autonomic, sensory, and visual functions. Women are more frequently affected than men and young adults are the primary demographic. Current research on demyelinating diseases has progressed substantially. Treatment strategies focus on preventing acute episodes and recurrence, with high-dose corticosteroids and intravenous immunoglobulin commonly used for acute CNS demyelinating disease episodes (Zorrilla Veloz et al., 2022). Emerging treatments, such as monoclonal antibodies targeting molecules like complement protein C5, provide new options but remain relatively scarce (Coggan et al., 2015; Fazakerley and Walker, 2003).

This article reviews PIEZO1, demyelinating diseases, and the role of PIEZO1 in these conditions. It discusses the structure, signaling mechanisms, pharmacological properties, and modulators of PIEZO1, and summarizes current knowledge of PIEZO1 channels. The article introduces the concepts, classifications, and etiologies of demyelinating diseases while highlighting PIEZO1’s role in the nervous system, particularly in myelin development and formation. Finally, it explores the therapeutic potential of targeting PIEZO1 in demyelinating diseases and its implications for expanding treatment strategies.

## 2 Overview of the PIEZO1 channel

### 2.1 Physiological role of PIEZO1

In 2010, Coste et al. (2010) discovered a protein essential for the expression of pressure-activated mechanically activated (MA) cation channels in the mouse neuroblastoma cell line. This protein is encoded by the Fam38A gene and is a member of the Piezo protein family, which includes PIEZO1 and PIEZO2. PIEZO1, found in eukaryotes, is a highly conserved mechanosensitive ion channel in the evolutionary process. A substantial body of research has demonstrated the important roles of PIEZO1 in vascular development (Baratchi et al., 2017), red blood cell volume regulation (Faucherre et al., 2014), and urine osmoregulation. In the CNS, PIEZO1 acts as a key regulator of neurodevelopment (Martins et al., 2016), involved in processes such as axon pathfinding (Koser et al., 2016), neural stem cell differentiation (Pathak et al., 2014), and the formation of CNS myelin (McHugh et al., 2010; O’Meara et al., 2011; Shimizu et al., 2017). Notably, studies have shown that pharmacological activation of PIEZO1 channels can induce demyelination while blocking PIEZO1 channels can prevent axonal and myelin damage in the CNS and also alleviate secondary progressive neurodegeneration following demyelinating diseases (Velasco-Estevez et al., 2020). These findings underscore that PIEZO1 is an important biomechanical sensor, playing a crucial role in mechanical signal transduction during various physiological and pathophysiological processes.

### 2.2 The structure and structural dynamics of PIEZO1

Compared to other ion channels, PIEZO proteins are remarkably large (composed of over 2,000 amino acids) and exhibit a unique topology (Coste et al., 2010). In 2018, Saotome et al. (2018), using cryo-electron microscopy (cryo-EM), analyzed the structure of the full-length mouse PIEZO1 channel, consisting of 2,547 residues, at improved resolutions of 3.8 Å and 3.97 Å compared to the previous 4.8 Å (Ge et al., 2015). This study provided more precise insights into the structural organization of PIEZO1 and offered a plausible explanation of its role in the mechanical transduction process.

**Figure 2:**
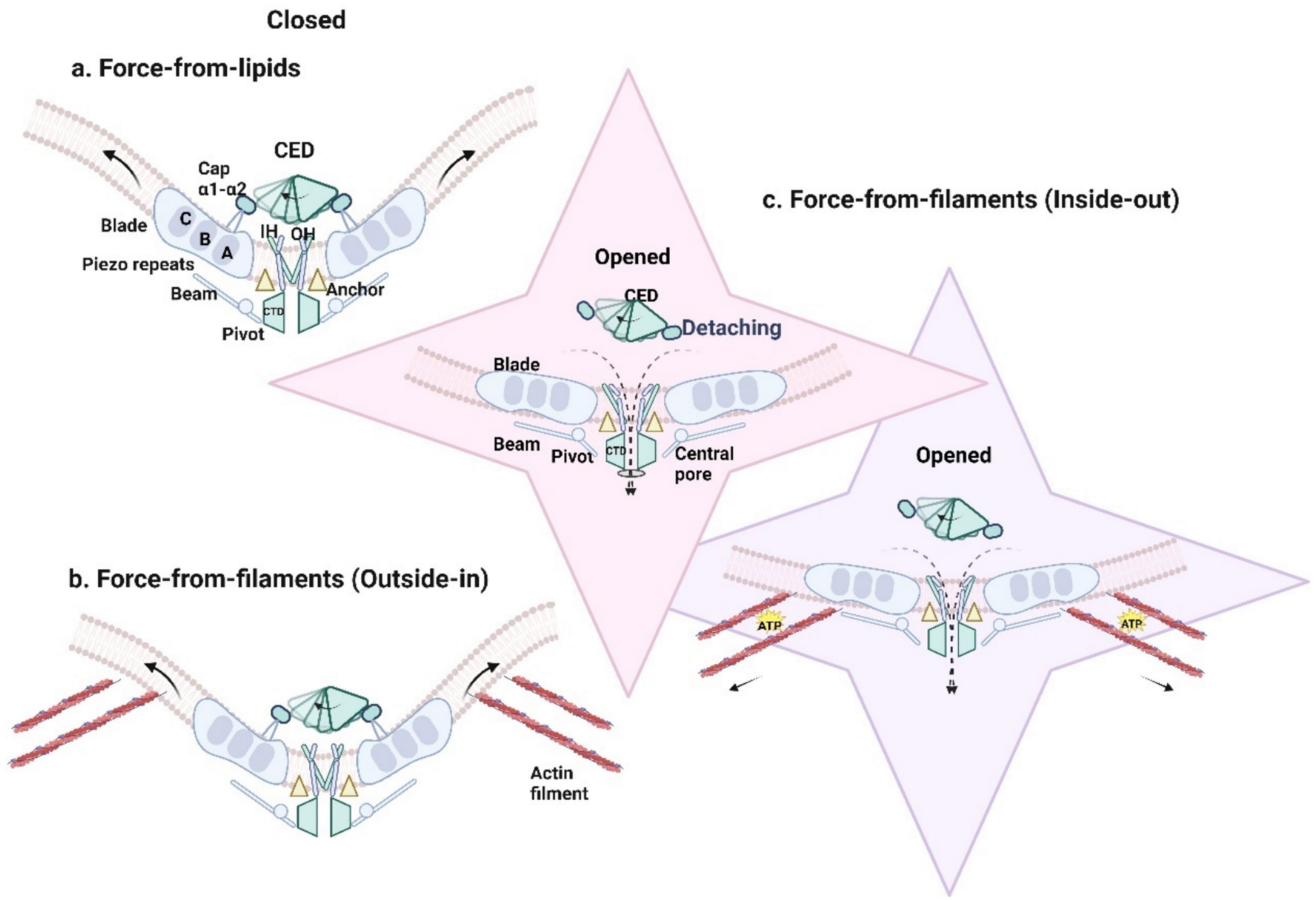
**(a)** Membrane tension directly gates PIEZO1 protein and results in the opening of the channel. Introduced by mechanical forces, the downward and tilting motion of THU8 resulted in the complete detachment of the cap from the blades, the clockwise rotation of the detached cap, the change of the three propeller blades from an outwardly curved state to a completely flattened state, and the opening of the central ion channel to generate mechanically activated cationic currents (black dotted line). **(b)** PIEZO1 responds to forces exerted on the cell such as membrane tension, shear stress substrate stiffness, substrate nano topology, and osmotic pressure in passive mechanical transduction. **(c)** PIEZO1 is activated by traction forces (solid black arrow) generated along the actin filament by the cell itself and ATP hydrolysis is required.

The PIEZO1 channel forms a homotrimer with a 38-transmembrane helical topology. This structure is curved and embedded in the lipid membrane (Wang et al., 2019). Functionally, the trimer can be divided into two distinct modules: the central ion-conducting pore module and the peripheral mechanical transduction module. The former mediates cation influx into the cytosol, while the latter senses mechanical signals (Ge et al., 2015; Guo and MacKinnon, 2017; Zhao et al., 2018) (Figure 2).

The central ion-conducting pore module comprises three parts: the extracellular cap domain (C-terminal extracellular domain, CED) (residues 2,189–2,547), the inner helix, and the cytosolic C-terminal intracellular domain (CTD) (Ge et al., 2015; Wang et al., 2019). In 2016, Zhao et al. (2018) first revealed the significance of the pore module (residues 2,189–2,547) in determining the unitary conductance and ion selectivity of mouse PIEZO1. The CED is flexibly connected to the transmembrane domains, potentially influencing pore characteristics across species and facilitating ion entry into the pore. Structural comparisons of PIEZO1 and PIEZO2, along with conformational changes during activation, identified a transmembrane constriction site as a potential gate. This gate, located between upper residue L2469 and lower residues V2476 and F2480, is closely associated with the displacement of the cap domain, which regulates mechanical sensitivity and ion permeability (Wang et al., 2019). The decoupling of the dome structure and leaflet dynamics is possible through a gate-controlled mechanism involving the central transmembrane pore (Yang X. et al., 2022). The downward and tilted movement of THU8 (transmembrane helical unit 8, TM29 to TM32) leads to complete detachment of the cap from the leaflet. The counterclockwise rotation of the detached cap and the outward movement of THU9 (TM32 to TM36) may jointly result in partial expansion of the inner and outer helices, thereby activating PIEZO1. By deleting the α1- and α2-helical domains of the cap or cross-linking the α1-helix of the cap with THU8, the mechanical activation of PIEZO1 can be eliminated (Wang et al., 2019; Yang X. et al., 2022; Lewis and Grandl, 2020).

Additionally, two lateral portals were identified between the inner helices. The two lateral portals formed by residues E2495 and E2496 of PIEZO1 may form an intracellular ion-permeable pathway (Wang et al., 2019; Zhao et al., 2018). The lateral plug gate (consisting of Trp1395-Thr1405) normally physically blocks the lateral portal and restricts the pathway for ion penetration. If a selective splice variant results in the absence of the side plug, the side portal is completely open and ions can permeate directly through the side portal, as shown by the enhanced mechanical sensitivity of PIEZO1.1 (deletion of residues 1,382–1,405 of PIEZO1), but reduced Ca^2+^ permeability (Geng et al., 2020). Notably, the side chains of residues M2493 and F2494 create a hydrophobic barrier at the cytosolic “neck, “playing a crucial role in the gating properties of PIEZO1 (Saotome et al., 2018).

The peripheral mechanical conduction mode is a unique three-blade propeller shape, and the propeller blades are composed of three four-transmembrane spiral bundles, which can move horizontally on the membrane plane through their peripheral areas, which is called piezoelectric repetition. The peripheral conduction mode consists of a curved transmembrane region, intracellular “beam” (long beam-like structures with residues L1342 and L1345 as fulcrums) and extracellular “anchors” (a triangular triple helix wedge is formed between inner and outer transmembrane helices and piezoelectric repetitive sequence A), and each precursor contains at least 26 transmembrane helices (Saotome et al., 2018; Ge et al., 2015).

The curved blades and beam-like structures, pivoting around residues L1342 and L1345, may function as a lever-like apparatus. This mechanical transduction mechanism likely enables PIEZO channels to translate large conformational changes in the peripheral blades into smaller movements at the central gate, regulating conformational changes caused by membrane tension and facilitating cation-selective permeability (Zhao et al., 2018; Yang X. et al., 2022; Lewis and Grandl, 2020). The “anchor” domains, connecting the inner and outer helices to the CTD plane, further support allosteric gating and stabilize the ion conduction pore (Saotome et al., 2018).

Lin et al. (2019) found that PIEZO1 reconstituted in lipid bilayers undergoes reversible flattening in correlation force-induced mechanical sensing, with leaflet flattening and simultaneous expansion of the in-plane membrane area, as probed by high-speed atomic force microscopy.

The extended blades of the PIEZO channel can locally deform the lipid bilayer into a dome-shaped structure. The PIEZO1 protein and the curved lipid bilayer surrounding the channel are collectively referred to as the PIEZO dome (Guo and MacKinnon, 2017). Haselwandter and MacKinnon (2018) introduced the concept of the “PIEZO membrane footprint” to describe the area of lipid bilayer deformation outside the dome, demonstrating that this footprint may amplify PIEZO1’s sensitivity to applied forces. Research by Yang et al. (2022) revealed that PIEZO1 not only induces curvature in the surrounding membrane but also directly responds to membrane tension associated with curvature changes, providing a precise quantitative description of curvature-based gating.

PIEZO1 exhibits a unique transmembrane helical topology and specific mechanical transduction components. Yang X. et al. (2022) observed that both the transmembrane gate and the lateral plug gate are expected to open in response to conformational changes through the movement of the extracellular cap and the intracellular beam of the mechanical sensing blade, thus translating the significant deformation of the blade into a moderate deformation of the transmembrane gate and the lateral plug gate (Xiao, 2024). These features confer high mechanical sensitivity and cation selectivity to PIEZO1.

### 2.3 Activation and deactivation of PIEZO1

The activation of PIEZO1 channels is primarily mediated through mechanical and chemical gating mechanisms. PIEZO1 exhibits exceptionally high mechanical sensitivity, responding sensitively to changes in membrane tension. The measured half-maximal activation tension (T50) is approximately 1.4 mN/m (equivalent to 1.4 pN/nm) (comparisons with T50 values of other channels could be added here) (Lewis and Grandl, 2015). The mechanical mechanisms driving PIEZO1 channel opening may involve two pathways: the “force-from-lipids” model mechanical transduction originating from the cell membrane and the “force-from-filaments” model (mechanical transduction via cytoskeletal tethers) (Murthy et al., 2017).

The “force-from-lipids” model suggests that mechanical forces on the cell membrane cause a reorganization of membrane lipids around the channel proteins, leading to the PIEZO1 channel opening (Arnadóttir and Chalfie, 2010). The mechanical disturbance of about 3.4mN/m (without other cell components) in the bilayer of droplet interface can open the recombinant mouse PIEZO1, which proves that PIEZO1 in the lipid bilayer of droplet can directly respond to the mechanical force in the lipid bilayer, but the experimental data does not rule out the possibility that other cell components such as cytoskeleton participate in the modulation of piezoelectric mechanical sensitivity (Syeda et al., 2016). Under mechanical force, repeated conformational changes in PIEZO1 are transmitted via the beam and/or anchor domains to the inner helix and CTD. This process causes the three propeller blades of PIEZO1 to transition from an outward-bent state to a fully flattened configuration (Figure 2a). Consequently, the central ion channel is reversibly opened on a sub-millisecond timescale, allowing cation influx and generating mechanically activated cation currents (Saotome et al., 2018; Yang X. et al., 2022; Romero et al., 2019).

**Figure 3:**
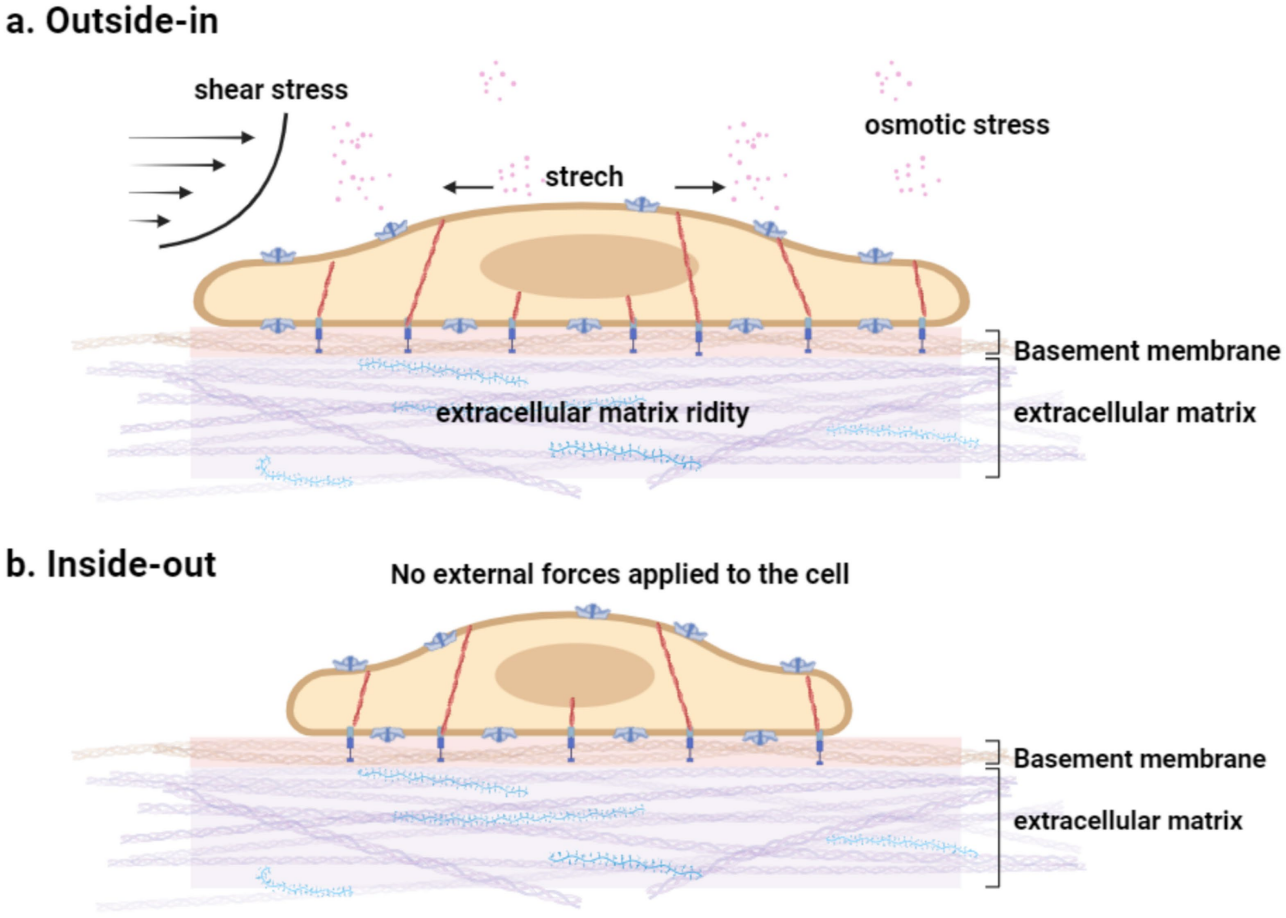
**(a)** Mechanical stimuli encountered by cells. Cells are subject to various dynamic mechanical stimuli in the environment and sense mechanical cues in the matrix. The cytoskeleton has a mechanical protective effect and contributes to the fine adjustment of PIEZO1 sensitivity in intact cells. **(b)** No external force stimulus is exerted on the cell.

In contrast, the “force-from-filaments” model posits that PIEZO1 channels are activated through interactions with the extracellular matrix or cytoskeletal proteins (Arnadóttir and Chalfie, 2010). Depending on the origin of the mechanical force—intracellular or extracellular—mechanical transduction can be categorized as either “outside-in” or “inside-out.” In “inside-out” mechanical transduction, cells actively generate traction forces using motor proteins to probe substrate stiffness (Figures 2c, 3b) (Nourse and Pathak, 2017). In “outside-in” mechanical transduction, cells passively respond to external forces such as indentation (Coste et al., 2010), membrane tension (Coste et al., 2010), shear stress (Li et al., 2014; Ranade et al., 2014), substrate stiffness (Pathak et al., 2014), substrate nano topography (Blumenthal et al., 2014), or osmotic pressure (Syeda et al., 2016) through mechanical reinforcement by the cytoskeleton (Figures 2b, 3a). PIEZO1 channels in the plasma membrane are activated and responsive to both “inside-out” and “outside-in” mechanical stimuli.

In the absence of externally applied mechanical forces, “inside-out” mechanical transduction underpins the spontaneous activity of PIEZO1: actively generated traction forces trigger channel activity, which can be suppressed by disrupting these forces (Pathak et al., 2014). The intact cytoskeleton provides essential mechanical support to the plasma membrane; experiments have demonstrated that disrupting actin polymerization using cytochalasin D reduces PIEZO1’s mechanical sensitivity (Gottlieb et al., 2012). However, the molecular mechanisms underlying the interplay between “outside-in” and “inside-out” mechanical transduction in PIEZO1 remain unclear.

Although PIEZO1 is primarily activated by mechanical signals, it can also be chemically activated, with chemical gating modulating its function. For example, Yoda1 (2-[5-[(2,6-dichlorophenyl)methyl]thio]-1,3,4-thiadiazol-2-yl-pyrazine) has been identified as an agonist of human and mouse PIEZO1 (Syeda et al., 2015). Two additional chemical activators, collectively termed Jedi1/2, have been found to induce dose-dependent responses specifically in cells transfected with mouse PIEZO1 (Wang et al., 2018). In concentration-response experiments, both Yoda1 and Jedi1/2 induced Ca2^+^ responses in human or mouse PIEZO1-transfected cells but not in PIEZO2-transfected cells, demonstrating their selectivity for PIEZO1 (Wang et al., 2018). Despite being PIEZO1 activators, Jedi1/2 and Yoda1 exhibit distinct activation mechanisms. Jedi1, characterized by greater hydrophilicity compared to Yoda1, enhances PIEZO1-mediated poking currents only when applied extracellularly. In contrast, intracellular application of Yoda1 also effectively enhances poking currents, with synergizing effects with extracellular Jedi1 application (Syeda et al., 2015; Wang et al., 2018). Furthermore, comparative studies suggest that Jedi and Yoda1 activate and regulate PIEZO1 by targeting different sites along the blade-beam gating pathway: Jedi acts upstream on the blade, while Yoda1 acts downstream on the beam (Wang et al., 2018; Qi et al., 2015).

GsMTx4 (Grammostola mechanotoxin #4), a gating modifier peptide derived from spider venom, can inhibit both PIEZO1 and PIEZO2 channels. Compared to other cysteine-knot (ICK) peptides isolated from spider venom, GsMTx4 exhibits significantly higher efficacy and non-stereospecific inhibition of mechanosensitive channels (Suchyna et al., 2000). GsMTx4 establishes a dynamic reservoir in the outer monolayer of the membrane in a tension-dependent manner, reducing the efficiency of force transmission between the lipid bilayer and the channel by altering the local tension distribution. This diminishes the effective magnitude of mechanical activation on the PIEZO1 channel (Gnanasambandam et al., 2017). A key difference between GsMTx4 and other ICK peptide inhibitors lie in GsMTx4’s relatively superficial binding to the monolayer, its enhanced penetration under membrane expansion, and its greater accessibility for transitioning to deeper states with increasing tension (Gnanasambandam et al., 2017). Additionally, gadolinium ions (Gd3+) at micromolar concentrations (Cinar et al., 2015) and the polycationic compound ruthenium red (RR) (Coste et al., 2012) can also inhibit PIEZO channels.

Studies suggest that beyond direct changes in membrane tension and the effects of known agonists and inhibitors, other extracellular and intracellular signals, such as membrane composition (Romero et al., 2019), the cytoskeleton (Saotome et al., 2018; Yang X. et al., 2022; Lewis and Grandl, 2015), membrane-altering proteins like STOML3 (Qi et al., 2015; Poole et al., 2014), and pH (Bae et al., 2015), can influence the activity of PIEZO1 channels. Under negative resting membrane potentials, the percentage of PIEZO1 channels readily activated is extremely low. Thus, altering the resting membrane potential can significantly increase the proportion of channels available for activation (Moroni et al., 2018).

### 2.4 The mechanism by which PIEZO1 conducts mechanical a signals/PIEZO1-mediated mechanical transduction

Mechanical signals activate the PIEZO1 channel, leading to the influx of extracellular cations such as Ca^2+^, Na^+^, and K^+^. This induces the propagation of electrical signals and initiates intracellular second messenger pathways. The downstream signaling pathways activated by PIEZO1 primarily include the following: Induction of ATP release and activation of purinergic P2 receptors: These receptors include ion channel P2X receptors and certain G-protein-coupled P2Y receptors (Wei et al., 2019). As a crucial autocrine or paracrine signaling molecule, ATP mediates intercellular communication by activating P2X and P2Y receptors. Studies support that PIEZO1 activation under mechanical stimulation can trigger ATP release. This ATP-mediated coupling between PIEZO1 and P2 receptors may represent a universal signaling mechanism (Cinar et al., 2015; Miyamoto et al., 2014; Peng et al., 2016; Wang et al., 2016). Activation of calpain: Research by Li et al. (2015) revealed that the downstream effect of PIEZO1-dependent Ca^2+^ signaling in vascular endothelial cells is the activation of calpain, adhesion remodeling, and spatial realignment of endothelial cell polarity under applied force. Liu et al. (2018) proposed that during immune synapse formation, calpain is activated downstream of PIEZO1-dependent Ca^2+^ signaling, thereby promoting TCR (T-cell receptor) signaling and facilitating the activation of autoreactive T cells. In summary, PIEZO1 is activated by sensing forces from the lipid membrane and is regulated by various chemical and environmental signals. It serves as a key modulator, converting mechanical forces into free-energy-dependent molecular conformational changes. PIEZO1 acts as a critical link between the extracellular physical environment and intracellular signaling cascades of different levels (Zheng et al., 2023).

## 3 The role of PIEZO1 in myelination and development

### 3.1 Distribution and expression of PIEZO1 in the nervous system

PIEZO1 is expressed in both neurons and glial cells; however, studies on laboratory mouse models indicate that PIEZO1 is primarily localized to neurons. María et al. investigated the expression of PIEZO1 channels in different brain regions of juvenile rats using coronal slices. Their results revealed relatively high expression levels of PIEZO1 channels in the optic nerve bundle of juvenile rats. Additionally, high expressions were observed in the corpus callosum axonal tracts, hippocampal fibers, and cerebellar dendrites (Jäntti et al., 2022; Velasco-Estevez et al., 2018).

Research has shown that neurons and glial cells exhibit a high degree of mechanical sensitivity. For example, Bollmann et al. (2015) found that microglia cultured on stiffness-gradient hydrogels migrate toward regions with higher stiffness, demonstrating a tendency for stiffness sensing. PIEZO1 channels play a role in neuronal mechanical sensation and axon guidance during brain development, enabling migrating growth cones to detect mechanical signals in their environment. As growth cones navigate through different brain regions toward their final targets, they likely receive continuous feedback on local ECM stiffness or the apparent density of the glial cell network. Furthermore, PIEZO1 serves as a regulator of physical communication between neurons and glial cells. *In vitro*, neurons grown on relatively smooth glass substrates interact with glial cells. Blocking PIEZO1 channels with the inhibitor GsMTx4 disrupts communication between neurons and glial cells (Velasco-Estevez et al., 2018).

Although PIEZO1 is predominantly distributed in neurons, its expression in glial cells also plays crucial physiological roles. For instance, activation of PIEZO1 channels in astrocytes can alter communication between astrocytes and neurons, influencing neuronal excitability (Csemer et al., 2024). PIEZO1 also contributes to Aβ plaque clearance in Alzheimer’s disease (AD) models. María et al. explored the expression of mechanosensitive PIEZO1 channels in response to amyloid pathology in aging AD rat models and found that Aβ plaques induced upregulation of PIEZO1 channels in astrocytes under aging conditions (Velasco-Estevez et al., 2018; Yu et al., 2023).

PIEZO1 is also involved in the protective role of microglia, the immune cells of the brain responsible for maintaining brain homeostasis, in AD. In both human and mouse AD models, activation of PIEZO1 by Yoda1 improves microglial phagocytosis, leading to enhanced Aβ clearance. The function of microglia surrounding plaques, particularly their motility and Aβ phagocytosis, may be key determinants in controlling AD pathology (Jäntti et al., 2022; Reich et al., 2018). These findings suggest that modulating PIEZO1 activation can improve impaired microglial function in AD, thereby reducing Aβ burden.

Additionally, PIEZO1 serves as a key mediator of mechanical transduction in oligodendrocyte progenitor cells (OPCs). Michael et al. demonstrated that PIEZO1 regulates cell numbers during central nervous system development, indicating that tissue stiffness is a critical regulator of OPC aging. To investigate the role of PIEZO1 in OPC activity, Michael et al. transfected PIEZO1 siRNA into aged rat OPCs and observed a 4-5-fold increase in proliferation and differentiation on stiff hydrogels compared to controls. Reduced PIEZO1 activity led to decreased sensitivity to mechanical signals (Navarrete et al., 2021). The study also found that both the PIEZO1 gene and protein expression significantly increase with age, and PIEZO1 is highly expressed in OPCs (Segel et al., 2019).

PIEZO channels are also the primary mechanosensitive channels in Schwann cells. Research by Brandon et al. using adeno-associated virus (AAV) vectors revealed that PIEZO1 in Schwann cells contributes to mechanical hypersensitivity following nerve injury. The YAP/TAZ co-transcription factors mediate Schwann cell responses to extracellular matrix stiffness or stretching (Tricaud, 2018; Acheta et al., 2022; Itson-Zoske et al., 2022). Furthermore, Jing et al. demonstrated that PIEZO1 regulates Schwann cell aging and promotes peripheral nerve fibrosis and scar formation (Liang et al., 2024; Grove et al., 2017; Piccolo et al., 2014).

Moreover, PIEZO1 regulates blood flow in the central nervous system (CNS) and functions as a mechanosensory channel in CNS vasculature, playing a critical role in controlling cerebral blood flow. In addition to participating in the mechanical sensitivity of nervous system cells and facilitating pathological clearance of substances like Aβ plaques, PIEZO1 activates a cascade of intracellular signaling events, including the contraction and relaxation of CNS capillaries. PIEZO1 channel activation induces calcium influx, which subsequently activates nitric oxide synthase (NOS), leading to the release of the vasodilator nitric oxide (NO) (Harraz et al., 2022; Cudmore and Santana, 2022; Zeng et al., 2018).

### 3.2 Composition and formation of myelin sheath

Myelin is a specialized multilayered cellular membrane that wraps around axons in the central nervous system (CNS) and peripheral nervous system (PNS), a structure unique to vertebrates. Myelin is primarily composed of myelin membranes, which are extensions of cellular membranes. Due to the high electrical resistance of myelin and the presence of tens to hundreds of these high-resistance layers, the multilayered compact myelin sheath ensures that myelinated segments of axons remain relatively insulated. This structure enables nerve impulses—electromagnetic waves driven by electrical potentials—to propagate rapidly along axons through saltatory conduction, increasing conduction velocity from less than 1 m/s to 50–100 m/s while reducing energy consumption during transmission. Moreover, myelin contributes to the plasticity of neural circuits, significantly influencing neural functions such as learning and memory (Salzer, 2015; Liu et al., 2019; Xin and Chan, 2020; Taylor and Monje, 2023; Kuhn et al., 2019; Bernardo and Visentin, 2023).

#### 3.2.1 Structural units of the myelin sheath

Myelin is produced by oligodendrocytes and Schwann cells and is composed of a lipid bilayer membrane, proteins embedded within it, and a small fraction of water. Lipids account for approximately 70–85% of myelin, primarily consisting of galactosyl ceramides, saturated long-chain fatty acids, and cholesterol, with cholesterol playing a crucial role in myelin assembly. The outer leaflet of the membrane contains specialized, dynamic, discrete domains known as lipid rafts, which are characterized by selective lipid concentrations and contribute significantly to myelin stability (Salzer, 2015; Kister and Kister, 2023; Siems et al., 2021).

Proteins make up about 15–30% of myelin and include myelin basic protein (MBP), myelin protein zero (P0), and proteolipid protein (PLP). MBP and P0 are critical for the formation of compact myelin. P0, a member of the immunoglobulin superfamily, is a transmembrane adhesion molecule that facilitates extracellular leaflet attachment through homophilic adhesion. MBP, a positively charged hydrophobic protein, interacts electrostatically with negatively charged lipid molecules, allowing it to “anchor” to the inner surface of the cell membrane. This interaction plays an essential role in myelin compaction and stability (Liu et al., 2019; Poitelon et al., 2020; Capodivento et al., 2024; Giussani et al., 2020; Boggs, 2006).

It is currently believed that myelin-forming cells generate a lamellar structure 132 the myelin sheath loosely envelops the axon. As the nervous system develops, myelin transcription levels rise, and myelin protein expression increases. Extracellular leaflet compaction occurs as P0 proteins on adjacent membranes interact in a tetrameric form, while cytoplasmic leaflet compaction is mediated by electrostatic interactions between MBP and the lipid bilayer, further stabilized by interactions with the cytoplasmic tail of P0. This process results in the formation of multilayered, compact myelin (Tognatta et al., 2020; Fields, 2014).

Under electron microscopy (EM), two alternating lines can be observed: the major dense line (MDL), representing the tight alignment of the cytoplasmic leaflets, and the intraperiod line, reflecting the alignment of the opposing extracellular leaflets. The MDL is the structural unit of compact myelin, where the two glial cell membranes are separated by only a few nanometers, with the cytoplasm filling the intervening space. The expression level of MBP is positively correlated with the formation of the MDL (Salzer, 2015; Bolino, 2021).

#### 3.2.2 The process of myelination

**Figure 4:**
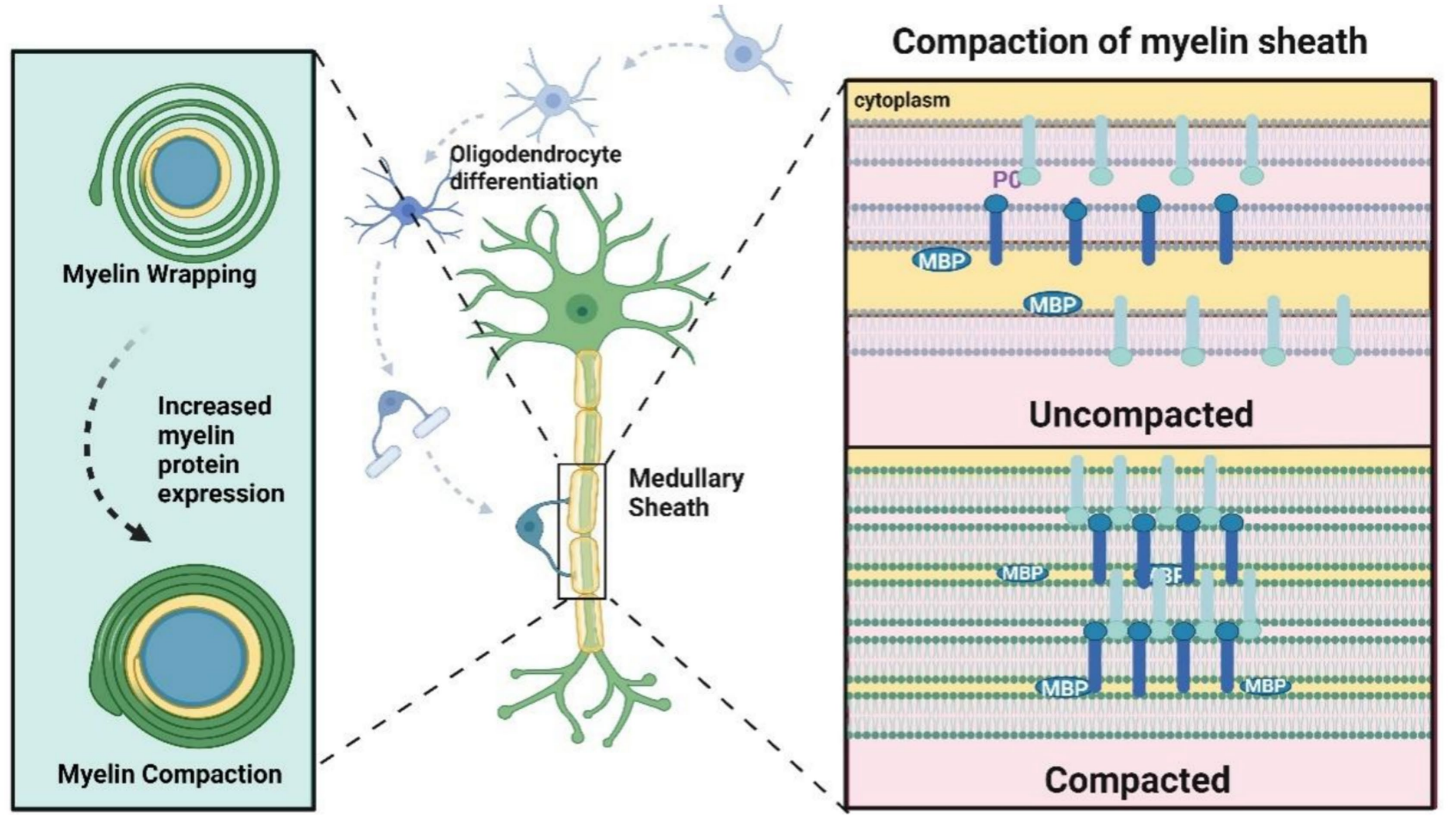
Formation of myelin sheath. The myelin sheath presents a loose spiral shape in the first few circles, followed by upregulation of myelin protein expression, including P0, MBP, etc., and the myelin sheath becomes tighter. Compaction “refers to the extracellular interaction between P0 tetramers on the cell membrane. The compression of cytoplasmic lobules is mediated by the electrostatic interaction between MBP and phospholipid bilayers, with the cytoplasmic tail being P0. The dense myelin sheath appears as a main dense line (MDL) (representing the tight attachment of cytoplasmic lobules) alternating with a periodic line (representing two attached extracellular lobules) on electron microscopy (EM).

The formation and compaction of myelin sheath are shown in Figure 4. One oligodendrocyte can form up to 100 myelin sheaths, whereas Schwann cells form myelin sheaths around single axons. Myelination is a highly regulated process, and in the peripheral nervous system (PNS), only axons with larger diameters undergo myelination. Specifically, Schwann cells typically form myelin around axons with diameters greater than 1 mm, a process known as “radial sorting.” Myelin formation is the result of myelinating cells spirally wrapping around axons. Initially, the myelin appears as a loose spiral in its early layers, after which myelin protein expression is upregulated, leading to tighter compaction of the sheath.

The radial sorting, wrapping, and elongation of Schwann cells along axons depend on the precise spatial and temporal remodeling of the actin cytoskeleton along the outer and inner membranes. F-actin is present in all cytoplasmic compartments of Schwann cells, and inhibiting actin assembly can block myelination in co-culture models. In contrast to the PNS, however, inhibiting myosin II appears to enhance myelination in the central nervous system (CNS), suggesting that the mechanisms of myelination differ between the PNS and CNS (Xin and Chan, 2020; Osso and Chan, 2017).

#### 3.2.3 The signaling that regulates myelination

The myelination process of Schwann cells is axon-dependent, as it is induced by axons. In contrast, oligodendrocytes can form myelin membranes even in the absence of axons. However, oligodendrocytes alone cannot fully replicate the complex myelination patterns observed in the central nervous system (CNS), as this process involves intricate mechanisms. Recent advancements have revealed that several regulatory signals play a role in this process.

##### 3.2.3.1 Exogenous signals

The exogenous signals that regulate myelination primarily originate from axonal Neuregulin-1 (NRG1) type III and laminin in the extracellular matrix. NRG1, a member of the epidermal growth factor (EGF) superfamily, transmits signals by binding to and activating members of the erbB tyrosine kinase receptor family, thereby regulating almost all aspects of Schwann cell lineage development. Research by Garratt et al. demonstrated that when NRG1 receptors are inactivated during the late stages of Schwann cell development, myelination in peripheral nerves is reduced, highlighting the essential role of NRG1 in the myelination process (Newbern and Birchmeier, 2010; El-Bazzal et al., 2023; Arthur-Farraj et al., 2011).

Laminin, synthesized by Schwann cells, acts as a key autocrine signal that is indispensable for myelination. This evidence was first proposed by Madrid et al. based on the phenotype of dystrophic mice, which exhibited basement membrane defects and abnormal clustering of myelinated spinal nerve roots. Additionally, co-culture experiments by Bunge et al. provided further support: in the absence of basement membrane assembly, Schwann cells were unable to form myelin. However, when laminin was added to the culture system, myelination was restored. Further research by Chen and Strickland revealed that knocking out laminin in Schwann cells leads to abnormalities in myelin sheath formation *in vivo* (Arthur-Farraj et al., 2011; Kim et al., 2017).

Collectively, these studies reveal that NRG1 and laminin are indispensable signaling regulators in the process of myelination.

##### 3.2.3.2 Endogenous signal

The endogenous signals that regulate myelination primarily involve three pathways: PI3K, PLC-γ, and mitogen-activated protein kinase (MAPK) (Martín-Durán and Hejnol, 2021). Studies conducted by Maurel and Salzer et al. have shown that the PI3K pathway (PI3K/Akt/mammalian target of rapamycin, mTOR) plays a critical role in Schwann cell development, including proliferation, survival, and myelination. Activation of the PI3K pathway enhances the ability of Schwann cells to ensheathe axons (Goebbels et al., 2010), while Sherman et al. found that the inactivation of mTOR in embryonic Schwann cells leads to thinner and shorter myelin sheaths (Zhang et al., 2021; Tiong et al., 2020).

The PLC-γ pathway is an important mediator of NRG1-related responses (Kao et al., 2009). The binding of NRG to the erbB co-receptor activates PLC-γ, initiating a series of reactions that drive the transcription of myelin protein. The MAPK pathway is critical for promoting early Schwann cell differentiation and is a key signal that mediates the myelination effects of NRG1. In a study by Sheean et al., the expression of the “activated” form of MAPK (Mek1) in Schwann cells resulted in a sustained increase in myelination (Ishii et al., 2021).

### 3.3 The effect of PIEZO1 on myelin

**Figure 5:**
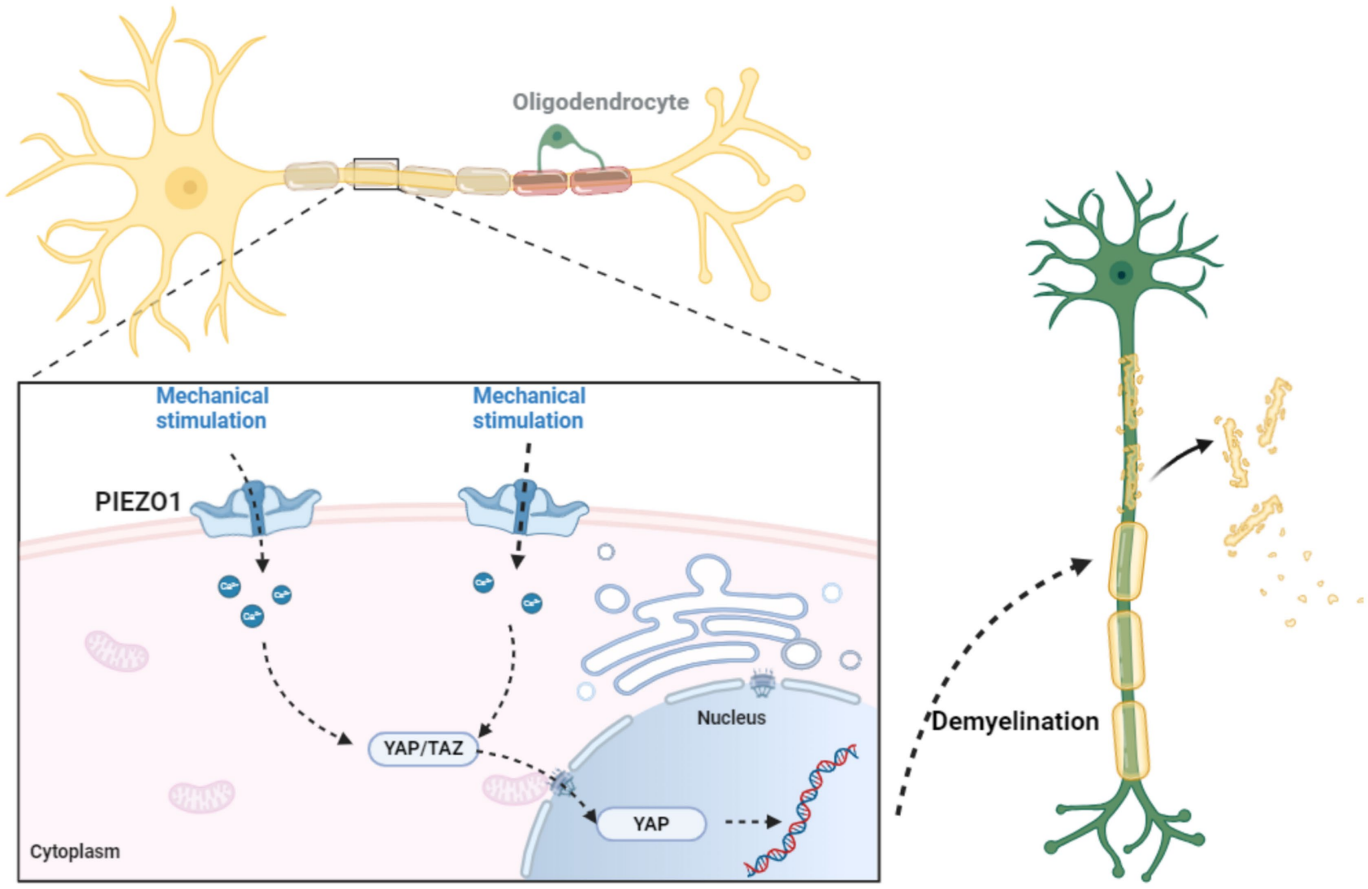
The role of PIEZO1 in myelin formation and regeneration. Research has shown that PIEZO1 in oligodendrocytes and Schwann cells plays a critical negative regulatory role in the formation and regeneration of myelin. PIEZO1 channel is the main mechanosensitive channel in Schwann cells. The activation of PIEZO1 channels on the cell membrane triggers transient calcium currents in oligodendrocytes, promoting the nuclear localization of transcriptional activators YAP/TAZ. YAP/TAZ will move to the nucleus, where they strongly induce cell proliferation. Therefore, the PIEZO1/YAP/TAZ pathway may be one of the hubs connecting external mechanical signals and cellular functions. The nuclear rejection of YAP/TAZ is a necessary condition for adult tissue homeostasis.

The role of PIEZO1 in myelin is mainly reflected in (1) Regulation of myelin formation and regeneration Studies have shown that PIEZO1 in oligodendrocytes and Schwann cells plays a key negative regulatory role in myelin formation and regeneration. The demyelination process involving PIEZO1 is shown in Figure 5. The PIEZO1 channel is the main mechanically sensitive channel in Schwann cells. Brandon et al.’s study using adenovirus (AAV) vectors to explore the role of PIEZO1 in peripheral nociception showed that PIEZO1 in Schwann cells contributes to mechanical hypersensitivity after nerve injury. The YAP/TAZ co-transcriptional factors are important regulators of peripheral nerve development and myelin maintenance and are signaling molecules sensitive to the stiffness or stretching of the extracellular matrix Schwann cells. The activity of YAP (Yes-associated protein) and TAZ (transcriptional coactivator with PDZ-binding motif) and the pathways they participate in affect cell proliferation, differentiation, and survival. YAP/TAZ exert their effects mainly by shuttling between the cell nucleus and cytoplasm Activation of the PIEZO1 channel on the cell membrane triggers a brief calcium current in oligodendrocytes, promoting the nuclear localization of the transcriptional activators YAP/TAZ. YAP/TAZ will move to the cell nucleus, where they strongly induce cell proliferation. Subsequently, YAPTAZ are inactivated by phosphorylation and degradation by the Hippo pathway. Therefore, the PIEZO1/YAP/TAZ pathway may be one of hubs connecting external mechanical signals to cellular functions. The nuclear exclusion of YAP/TAZ is a necessary condition for adult tissue homeostasis (Zheng et al., 2023; Acheta et al., 2022; Grove et al., 2017; Yang K. et al., 2022; Li H.-X. et al., 2021; Xu Z. et al., 2021; Knecht et al., 2021; Deng et al., 2022; Grove et al., 2024; Hu et al., 2022). Additionally, Liang’s et al. (2024) study demonstrated that PIEZO1 can induce the senescence of Schwann cells and promote the formation of fibrotic scars in peripheral nerve fibers. (2) Inhibition of PIEZO1 alleviates demyelination after intracerebral hemorrhage. Intracerebral hemorrhage (ICH) is a devastating form of stroke with high morbidity and mortality. The pathophysiology of ICH includes primary injury from mechanical stress caused the hematoma and secondary injury from the metabolic breakdown of blood. Previous studies in a mechanical micro balloon rat model have shown that the mass effect of hematoma can directly cause white matter injury, and that certain mechanosensitive channels may be involved in the pathophysiology of this phenomenon. After ICH, the expression PIEZO1 in oligodendrocytes increases. Jie et al. established a mouse model of ICH by injecting autologous blood into the basal ganglia and found that PIEZO1 was highly expressed within 48 h after ICH, mainly in oligodendrocytes. Then by intraperitoneal injection of Dooku1 to inhibit PIEZO1, they found that it significantly alleviated brain edema, myelin loss, androsis of injured tissues, significantly reduced apoptosis of oligodendrocytes, and significantly improved neurological function. Inhibiting PIEZO1 can also reduce the degradation of myelin basic protein, and thus improve neurological function after ICH. Jie’s team believes that the mechanism for this improvement in neurological is that Dooku1-mediated inhibition of PIEZO1 reduces intracellular endoplasmic reticulum stress and cell apoptosis via the PERK-AT4-CHOP and inositol-requiring enzyme 1 (IRE1) signaling pathways (Qu et al., 2022; Magid-Bernstein et al., 2022; Jin et al., 2024; Qi et al., 2024).

## 4 Demyelinating diseases

Demyelinating diseases are neurological disorders caused by the loss of myelin—a key component in the formation of myelin sheaths—or by damage to myelin-producing cells. The lost myelin is phagocytosed by macrophages. This demyelination disrupts signal conduction along axons, which can lead to axonal degeneration and neurological dysfunction. Since the myelin produced by oligodendrocytes in the central nervous system (CNS) and Schwann cells in the peripheral nervous system (PNS) have different chemical and immunological properties, demyelinating diseases can affect either the CNS or the PNS. Based on the affected location, these diseases are classified into CNS-demyelinating diseases and PNS-demyelinating diseases (Bernardo and Visentin, 2023; Kamil et al., 2019).

CNS demyelinating diseases can be categorized by their pathogenesis into the following types: inflammatory demyelination, viral demyelination, metabolic disorder-induced demyelination, hypoxic-ischemic demyelination, and focal pressure-induced demyelination. Oligodendrocytes, the myelin-forming cells in the CNS, are particularly vulnerable to oxidative stress due to their low antioxidant capacity and high intracellular iron content necessary for enzymatic activity. The most common pathological causes of oligodendrocyte damage in CNS include trauma, ischemia, or autoimmune attacks. These cells are highly fragile, and injuries such as trauma, immune-mediated attacks, or ischemia can lead to oligodendrocyte death and demyelination, subsequently triggering neurodegeneration. During the process of myelination, oligodendrocytes are susceptible to cytotoxic products, which impair their function. In demyelinating diseases, the death of oligodendrocytes can lead to extensive demyelination (Love, 2006; Kuhn et al., 2019; Jäkel et al., 2019).

**Figure 6:**
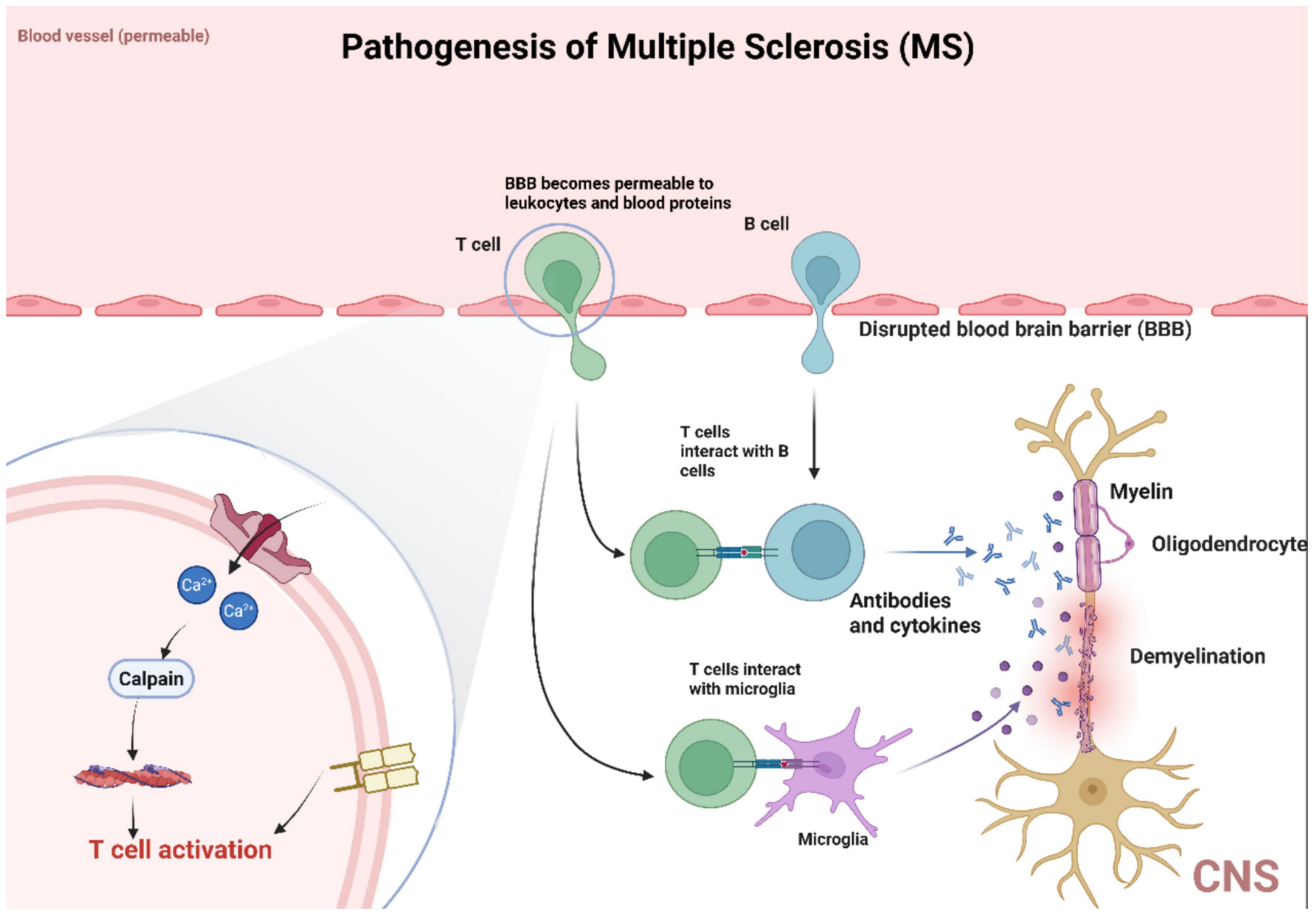
PIEZO1 is involved in MS pathology. The activation of PIEZO1 channels in axons negatively regulates myelination in the central nervous system, inhibiting PIEZO1 in CD4^+^ T cells and/or regulatory T cells (Tregs) to alleviate demyelination symptoms. PIEZO1 participates in the pathological process of MS by activating T cells. Multiple sclerosis (MS) is the most common demyelinating disease of the central nervous system, and its pathogenesis is believed to be an autoimmune disease caused by abnormal immune responses mediated by T cells (especially CD4^+^ T cells) in genetically susceptible individuals exposed to exogenous antigens. It is worth noting that B cells also play a crucial role in the pathogenesis of MS. Therefore, the characteristic of MS is immune-mediated damage to myelin sheath and oligodendrocytes, leading to myelin sheath destruction and oligodendrocyte death. When myelin is disrupted, it can separate from the surrounding axons. Axons without myelin sheaths become unstable and prone to neurodegeneration.

Multiple sclerosis (MS) is the most common CNS demyelinating disease, characterized by inflammation and demyelination. The prevailing view on its pathogenesis suggests that MS is an autoimmune disease. It is believed to result from an abnormal immune response mediated by T cells (especially CD4^+^ T cells) in genetically predisposed individuals exposed to foreign antigens. The role of PIEZO1 in MS is shown in Figure 6. Notably, research by Houser and colleagues indicates that B cells also play a crucial role in the pathogenesis of MS. Thus, MS is characterized by immune-mediated damage to myelin and oligodendrocytes, leading to myelin destruction and oligodendrocyte death. When myelin is destroyed, it can detach from the axons it surrounds. The axons left without myelin sheaths, become unstable and prone to neurodegeneration. In MS pathology, detached myelin components can form plaques of varying sizes throughout the CNS. These plaques are most easily identified in white matter, which is a prominent site of demyelination and inflammation. MS plaques can be classified into active plaques, inactive plaques, chronic active plaques, and shadow plaques. MS can be divided into (1) classic multiple sclerosis; (2) acute (Marburg type) multiple sclerosis, a rare fulminant variant; (3) optic neuromyelitis (Dewick disease), which usually causes extensive demyelination of affected segments of the optic nerve and spinal cord. Most patients present with vision loss, followed by paraplegia and sensory loss; (4) concentric sclerosis (Balo sclerosis), a rare disease characterized by alternating bands of demyelinating and myelinated white material, forming concentric rings or irregular stripes (Haki et al., 2024; Axisa and Hafler, 2016; Garg and Smith, 2015; Wang et al., 2023; Ruiz et al., 2019).

Clinically, MS presents with diverse symptoms, including weakness, paresthesia or focal sensory loss, optic neuritis, diplopia, ataxia, and vertigo. In addition to MS, other inflammatory demyelinating diseases include acute disseminated encephalomyelitis (ADEM) and acute hemorrhagic leukoencephalitis. ADEM predominantly affects children and usually occurs within 3 weeks of an infection, vaccination, or medication. It is caused by a T-cell-mediated hypersensitivity reaction. Acute hemorrhagic leukoencephalitis, a rare and fatal disease, is considered a hyperacute variant of ADEM (Reich et al., 2018; Martinsen and Kursula, 2021; Samadzadeh et al., 2023; Koike and Katsuno, 2021; Manjaly et al., 2019).

PNS demyelinating diseases (PDDs) are classified as acquired and hereditary types. Acquired PDDs include Guillain–Barré syndrome (GBS), chronic inflammatory demyelinating polyneuropathy, anti-myelin-associated glycoprotein (MAG) neuropathy, and POEMS syndrome. The primary hereditary PDD is Charcot–Marie–Tooth disease. GBS is the leading cause of acute neuromuscular paralysis worldwide and is characterized by tingling sensations, progressive weakness, autonomic dysfunction, and pain. GBS is also an immune-mediated disorder, where foreign antigens—most commonly lip oligosaccharides from *Campylobacter jejuni*—induce cross-reactive antibodies. These antibodies mimic host glycans via molecular mimicry and bind to ganglioside-like structures in peripheral nerves, resulting in damage to axons and Schwann cells (Kamil et al., 2019; Bellanti and Rinaldi, 2024; Finsterer, 2022; Sreelakshmi et al., 2024; Rodríguez et al., 2018).

## 5 The role of PIEZO1 in various demyelinating diseases

PIEZO1 appears to have a direct role not only in myelin formation and degradation but also in modulating immune responses associated with multiple sclerosis (MS). Studies have shown that in a mouse model of MS, experimental autoimmune encephalomyelitis (EAE), mice with PIEZO1-deficient T cells exhibited milder disease progression, along with increased TGF-β signaling and an overall increase in regulatory T cell (Treg) numbers. Furthermore, mice with PIEZO1-knockout Treg cells also demonstrated reduced EAE severity and enhanced Treg functionality. The role of PIEZO1 in MS can be summarized into three main aspects: demyelination and axonal degeneration, activation of macrophage and oligodendrocyte inflammation, and involvement in T cell receptor (TCR) activation (Radandish et al., 2021).

Demyelination and axonal degeneration are key pathological features of MS. Myelin is essential for rapid axonal conduction, and its loss typically disrupts neuronal communication. Additionally, myelin provides a conduit for energy transfer from oligodendrocytes to neurons. In the central nervous system (CNS), oligodendrocytes and axons are closely connected, facilitating crosstalk between neurons and oligodendrocytes (Velasco-Estevez et al., 2022). The homeostasis of neurons and oligodendrocytes is interdependent: axonal injury leads to demyelination, while demyelination can trigger neurodegeneration (Yang K. et al., 2022). Studies have shown that activation of axonal PIEZO1 channels negatively regulates myelination in the CNS (Acheta et al., 2022). While PIEZO1 is highly expressed in neurons, it is not expressed in mature oligodendrocytes. Yoda-1, a positive modulator of PIEZO1 channel opening, can induce demyelination. Conversely, the PIEZO1 antagonist GsMTx4 can mitigate psychosine (a cytotoxic lipid)-induced demyelination in ex vivo mouses cerebellar slice cultures and Lys phosphatidylcholine (LPC)-induced demyelination models (Csemer et al., 2024). Additionally, PIEZO1 activation inhibits axonal regeneration. After the axonal injury, PIEZO1 is recruited to the growth cone and activated, inducing calcium (Ca^2+^) transients and activating Ca^2+^/calmodulin-dependent protein kinase II (CaMKII). This leads to the activation of nitric oxide synthase (NOS) and downstream cGMP-dependent protein kinase (PKG) signaling, which limits axonal regeneration (Figure 3b) (Zheng et al., 2023; Csemer et al., 2024; Song et al., 2019; Li F. et al., 2021).

Macrophages and microglia are innate immune cells with diverse functions, including homeostasis, pathogen defense, and responses to injury. In LPC-induced demyelination mouse models, anti-inflammatory phenotypes favor oligodendrocyte differentiation and promote remyelination (Tricaud, 2018). Recent studies have investigated the role of PIEZO1 in macrophage function. Mechanical forces activate PIEZO1 in macrophages, promoting inflammation. Exposure to mechanical stress upregulates pro-inflammatory gene expression in bone marrow-derived macrophages (BMDMs), many of which are targets of hypoxia-inducible factor-1α (HIF-1α), requiring PIEZO1 activation. PIEZO1 also plays a role in macrophage polarization and in sensing microenvironment stiffness. In BMDMs, PIEZO1 enhances lipopolysaccharide (LPS)-induced inflammation while reducing interleukin-4 (IL-4)- and IL-13-induced healing responses. Additionally, PIEZO1 regulates macrophage function through microenvironment stiffness *in vitro* and influences immune responses to biomaterials implanted subcutaneously *in vivo*.

Typically, interactions between T cell receptors (TCRs) and peptides on MHC molecules trigger T cell activation. The strength of this interaction determines the extent of T-cell activation. Recent research indicates that mechanical forces contribute to optimal T cell activation, suggesting that the mechanosensitive ion channel PIEZO1 is involved in human T cell activation. In the absence of PIEZO1, immobilized crosslinking antibodies fail to induce maximal TCR activation in CD4^+^ and CD8^+^ T cells, a deficiency that can be rescued through chemical activation of PIEZO1 by Yoda-1 (Tricaud, 2018).

## 6 Using PIEZO1 as a target for demyelinating diseases

### 6.1 Current research on PIEZO1 as a target for demyelinating diseases

In recent years, numerous studies have suggested that PIEZO1 is a promising therapeutic target for demyelinating diseases. Velasco-Estevez et al. demonstrated that the PIEZO1 antagonist GsMTx4 can mitigate demyelination induced by the cytotoxic lipid psychosine, whereas pharmacological activation of PIEZO1 can induce demyelination (Velasco-Estevez et al., 2020). GsMTx4 has been shown to reverse astrocyte cell death and promote astrocytic hypertrophy (Velasco-Estevez et al., 2020). Astrocytes play a critical role in tissue repair and remyelination following central nervous system (CNS) injury. If astrocytic hypertrophy regresses without forming chronic glial scars, it may accelerate the repair of nerve damage (Su et al., 2009; Sofroniew, 2009). However, the use of GsMTx4 in vitro cannot prevent psychosine-mediated astrocyte toxicity from damaging neurons, presumably because PIEZO1 is primarily expressed in mature neurons and is less expressed in neural progenitor cells (NPCs) or immature astrocytes (Velasco-Estevez et al., 2020). Importantly, Velasco-Estevez et al’s. (2020) study demonstrated that GsMTx4 can prevent demyelination and neuronal damage induced by *in vivo* cortical injections of lysophosphatidylcholine (LPC). These findings suggest that blocking PIEZO1 channels with GsMTx4 may represent a feasible therapeutic strategy for CNS demyelinating diseases (Velasco-Estevez et al., 2020).

An experimental study on demyelination following intracerebral hemorrhage (ICH) found that PIEZO1 expression significantly increased 3 h post-ICH injury. Intraperitoneal injection of a moderate dose (10 mg/kg per mouse) of the PIEZO1 antagonist Dooku1 significantly reduced oligodendrocyte apoptosis, alleviated myelin degradation and mitigated both neuronal and myelin damage by inhibiting the unfolded protein response (Qu et al., 2022). Inhibiting PIEZO1 also blocked the eIF2α-PERK-ATF4 pathway, alleviating endoplasmic reticulum stress and apoptosis induced by ICH, thereby protecting myelin integrity (Qu et al., 2022). These findings indicate that PIEZO1 could serve as a potential therapeutic target for improving demyelination following ICH (Qu et al., 2022).

Yang K. et al. (2022) discovered that activation of PIEZO1 channels in axons negatively regulates myelination in the CNS. Inhibition of PIEZO1 in CD4^+^ T cells and/or regulatory T cells (Tregs) alleviated symptoms of experimental autoimmune encephalomyelitis (EAE). This suggests that PIEZO1 may be a future therapeutic target for multiple sclerosis treatment (Yang K. et al., 2022).

Sepsis-associated encephalopathy (SAE) can lead to oligodendrocyte damage and cognitive dysfunction via microglial activation. Xie et al. found that PIEZO1 knockout effectively reduced hippocampal oligodendrocyte demyelination in SAE mice (Xie et al., 2024). Targeting PIEZO1 with siRNA effectively reduced the secretion of inflammatory mediators, such as C-C motif chemokine 25 (CCL25) and anti-IL-18, by inhibiting the p38 (Mitogen-activated protein kinase) pathway. It also prevented oligodendrocyte ferroptosis by modulating the CCL25/GPR78 axis (Xie et al., 2024). These findings suggest that targeting PIEZO1 in microglia could represent a novel approach for addressing demyelinating diseases and related cognitive dysfunction induced by SAE (Xie et al., 2024).

### 6.2 A summary of these studies, limitations, challenges, and controversies

It can be observed that current research targeting PIEZO1 for the treatment of demyelinating diseases primarily involves directly inhibiting PIEZO1 using inhibitors, particularly GsMTx4. The advantage of this approach lies in its strong existing foundation, as research on GsMTx4 is already well-established. Further studies in this direction can thus minimize interfering factors and achieve high accuracy and feasibility. However, given that both PIEZO1 activators and inhibitors exhibit low affinity for PIEZO1, as well as poor solubility and stability, their pharmacological efficacy in the human body may be limited. Therefore, identifying alternative PIEZO1 modulators with improved pharmacological efficacy is crucial for advancing targeted therapies for demyelinating diseases.

The aforementioned studies primarily focus on inhibiting PIEZO1 to explore therapies for demyelinating diseases based on molecular interactions. Moving forward, we hope to see more research centered on PIEZO1 itself. For instance, studies could investigate whether modifying the structure of PIEZO1 can control the opening and closing of this channel, thereby regulating a series of physiological or pathological processes in the body. Alternatively, research could focus on the expression of PIEZO1 genes, targeting its DNA or RNA to influence its expression within the body. Additionally, attention could be directed to the signaling pathways involving PIEZO1. By activating or inhibiting specific steps or multiple components within these pathways, it may be possible to trigger a cascade of physiological responses.

Compared to the current research on the interaction between PIEZO1 and its inhibitors or activators, these types of studies are relatively limited. They pose greater challenges in terms of experimental feasibility and operability, and their outcomes may remain controversial. Nevertheless, these studies place greater emphasis on the intrinsic characteristics of the PIEZO1 molecule. By targeting its structure, gene expression, or involvement in signaling pathways, these approaches have the potential to address the pathological processes involving PIEZO1 in demyelinating diseases at a more fundamental level.

## 7 Conclusion

Demyelinating diseases refer to a group of disorders in which the myelin sheath is damaged due to various causes, leading to pathological changes in demyelination and subsequent impairment of the nervous system’s function. These diseases can affect individuals across all age groups, and the susceptible population may vary depending on the underlying cause. For instance, ischemic-related demyelination is more common in middle-aged and elderly individuals, while immune-and inflammation-related damage is more prevalent in children and young adults. The prognosis of demyelinating diseases varies significantly depending on the cause and the extent of the lesions. Some acute conditions can achieve full recovery with timely diagnosis and treatment, while chronic cases may result in lasting neurological deficits. In severe or critical cases, the disease can even pose a life-threatening risk.

PIEZO1 is expressed in multiple cells types and plays a role in sensing and responding to mechanical forces. It is expressed in certain types of neural cells and may be involved in mechanical transduction. During myelination in the peripheral nervous system, PIEZO1 can inhibit the longitudinal and radial formation of myelin sheaths. Recent studies have demonstrated that the inhibition of PIEZO1 can mitigate demyelination induced by psychosine and lipopolysaccharides. In models of cerebral hemorrhage, suppressing PIEZO1 alleviates endoplasmic reticulum stress and apoptosis in oligodendrocytes, thereby protecting myelin and improving neurological function after hemorrhage. PIEZO1 plays a crucial role in mechanical sensation within the central nervous system, and its dysfunction may be associated with the onset and progression of various neurological disorders, including demyelinating diseases.

In summary, PIEZO1 plays a pivotal role in demyelinating diseases, and its inhibition or activation may hold significant therapeutic and neuroprotective potential. Future research is expected to further elucidate the specific mechanisms of PIEZO1 in demyelinating diseases. More studies are needed to explore how PIEZO1 can be targeted for therapeutic intervention, paving the way for novel treatments for demyelinating diseases. Investigating the structure, gene expression, or signaling pathways of PIEZO1 may serve as valuable research directions, providing new insights and strategies for the treatment of demyelinating disorders.
